# Controlling aflatoxin contamination and propagation of *Aspergillus flavus* by a soy-fermenting *Aspergillus oryzae* strain

**DOI:** 10.1038/s41598-018-35246-1

**Published:** 2018-11-15

**Authors:** Ahmad F. Alshannaq, John G. Gibbons, Mi-Kyung Lee, Kap-Hoon Han, Seung-Beom Hong, Jae-Hyuk Yu

**Affiliations:** 1 Department of Food Science, University of Wisconsin-Madison, 1605 Linden Dr, Madison, WI 53706 USA; 2 Food Research Institute, University of Wisconsin-Madison, 1550 Linden Drive, Madison, WI 53706 USA; 3 Department of Food Science, University of Massachusetts, 240 Chenoweth Laboratory, 102 Holdsworth Way, Amherst, MA 01003 USA; 4 Biological resource center, Korea Research Institute of Bioscience and Biotechnology, 181 Ipsin-gil, Jeongeup-si, Jeollabuk-do 56212 Republic of Korea; 5 Department of Pharmaceutical Engineering, Woosuk University, Wanju, 55338 Republic of Korea; 6 Korean Agricultural Culture Collection, Agricultural Microbiology Division, NAS, RDA, Wanju, Republic of Korea; 7 Department of Bacteriology, University of Wisconsin-Madison, 1550 Linden Drive, Madison, WI 53706 USA; 8 Department of Systems Biotechnology, Konkuk University, Seoul, Republic of Korea

## Abstract

Aflatoxins (AFs) are a group of carcinogenic and immunosuppressive mycotoxins that threaten global food safety. Globally, over 4.5 billion people are exposed to unmonitored levels of AFs. *Aspergillus flavus* is the major source of AF contamination in agricultural crops. One approach to reduce levels of AFs in agricultural commodities is to apply a non-aflatoxigenic competitor, e.g., Afla-Guard, to crop fields. In this study, we demonstrate that the food fermenting *Aspergillus oryzae* M2040 strain, isolated from Korean Meju (a brick of dry-fermented soybeans), can inhibit aflatoxin B1 (AFB1) production and proliferation of toxigenic *A*. *flavus* in lab culture conditions and peanuts. In peanuts, 1% inoculation level of *A*. *oryzae* M2040 could effectively displace the toxigenic *A*. *flavus* and inhibit AFB1 production. Moreover, cell-free culture filtrate of *A*. *oryzae* M2040 effectively inhibited AFB1 production and *A*. *flavus* growth, suggesting *A*. *oryzae* M2040 secretes inhibitory compounds. Whole genome-based comparative analyses indicate that the *A*. *oryzae* M2040 and Afla-Guard genomes are 37.9 and 36.4 Mbp, respectively, with each genome containing ~100 lineage specific genes. Our study establishes the idea of using *A*. *oryzae* and/or its cell-free culture fermentate as a potent biocontrol agent to control *A*. *flavus* propagation and AF contamination.

## Introduction

**Figure 1:**
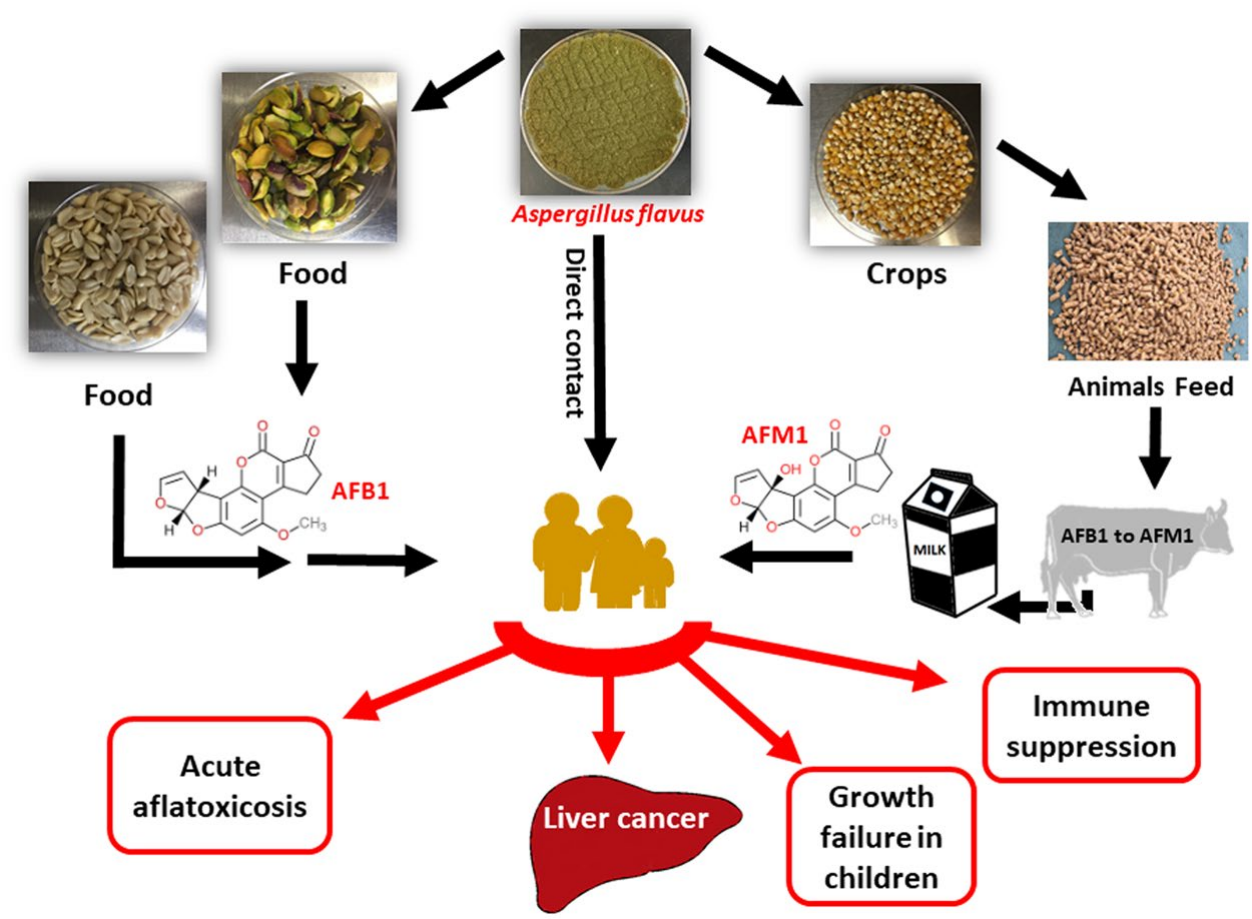
Schematic presentation summarizing the major AFB1 and AFM1 contamination/exposure routes and adverse health effects to human.

Aflatoxins (AFs) are a group of small molecular weight fungal toxins that threaten world food safety by contaminating ~25% of the world’s crops^1^. AFs are considered to be an unavoidable contaminant in human food and animal feed by the US Food and Drug Administration (FDA)^2^. Among AFs, aflatoxin B1 (AFB1) is the most potent carcinogen present in nature and is produced mainly by the ubiquitous soil filamentous fungus *Aspergillus flavus*^3^. AFs are acutely toxic, carcinogenic, mutagenic, teratogenic, and immunosuppressive, and are classified as group 1 carcinogens in human (Fig. 1)^4^. Of the 550,000–600,000 new liver cancer cases worldwide each year, it is estimated that 25,200–155,000 may be attributed to AF exposure^5,6^. Due to their high toxicity and carcinogenicity, over 120 countries have set maximum limits of AFs in foods (4~30 ppb) and feeds (20~300 ppb)^1^. In the U.S. corn industry, AF contamination could cause losses ranging from $52.1 million to $1.68 billion annually as was reported in year 2012^7^.

AFs can frequently contaminate cereals, oilseeds, spices, tree nuts, corn, groundnuts (peanuts), pistachios, chilies, black pepper, dried fruit and fig, raising global health and economy concerns^1^. Furthermore, animal by-products such as milk, meat, and egg can be indirect sources of AF exposure^8,9^. Human milk can have aflatoxin M1 (AFM1), a hydroxylated metabolite of AFB1, and pose a serious threat for infants^10,11^ (Fig. 1). In certain areas in Africa and Asia, AFs are considered as the leading cause of serious acute illnesses and deaths each year^12^. Improper storage of the crops, nuts, and grains further contributes to increased levels of AFs. Unfortunately, over half of the global population is exposed to high, unmonitored levels of AFs^13^.

Previous research has demonstrated that both water availability and temperature affect *A*. *flavus* growth and the expression of genes in the AF biosynthesis gene cluster^14^. Predictions associated with global warming suggest that *A*. *flavus* is likely to infect more crop plants, and will show increase expression of the AF biosynthetic genes (e.g., *aflD* and *aflR*)^15^, enhancing the risk of crop contamination by AF. With a 2 °C temperature increase, AFB1 is predicted to become a food safety issue in the European maize production^16^. Several strategies have been developed to reduce AF contamination, including the use of a non-toxigenic strain of *A*. *flavus* to outcompete and displace toxigenic strains^17,18^. This effective biological control method results in greatly reduced AF levels in a diversity of harvested agricultural products and has been applied worldwide^19,20^. Commercially available non-toxigenic *A*. *flavus* isolates include K49 (NRRL 30797, isolated from Maize), Afla-Guard (NRRL 21882, isolated from peanuts), and AF36 (NRRL 18543, isolated from Cottonseed)^21^.

Here, we investigated the potential of using *Aspergillus oryzae*, the food grade non-toxigenic domesticated ecotype of *A*. *flavus*, as a biocontrol agent for inhibiting AFB1 production and growth of the toxigenic *A*. *flavus* strain NRRL 3357. *A*. *oryzae* is used for food fermentation (*e*.*g*. sake, miso, soy sauce, meju) and is classified as a Generally Recognized As Safe (GRAS) organism by the FDA and the WHO^22,23^. Fermented soy pastes produced with *A*. *oryzae* are rarely contaminated with AFs. Thus, we hypothesize that there is a strong anti-mycotic potential of *A*. *oryzae* to outcompete *A*. *flavus* in soy-based food. In this study, we have found that *A*. *oryzae* M2040 (designated as M2040 hereafter) isolated from Korean Meju (a soy brick used to make soybean paste called Doen-Jang in Korea) inhibits growth and AFB1 production by *A*. *flavus* significantly better than the widely used commercial biocontrol isolate Afla-Guard. To quantify the competitive effects of M2040, we generated a GFP-labeled *A*. *flavus* NRRL 3357 strain and used to quantify the competitive displacement of *A*. *flavus* by M2040 in peanuts. Importantly, inoculum level of M2040 as low as 1% was effective for controlling of AFB1 production and *A*. *flavus* proliferation. Additionally, cell-free culture filtrate of M2040 grown in potato dextrose broth (PDB) inhibited *A*. *flavus* germination, propagation, and AFB1 production, suggesting the presence of anti-mycotic compound(s) in the M2040 fermentate. Whole genome sequencing and comparative analyses revealed the presence of an additional 1.5 Mbp in the M2040 genome (37.9 Mbp) compared to Afla-Guard (36.4 Mbp). We identified 111 M2040 lineage specific genes arranged in several clusters that may play a role in the observed phenotypes. This report provides a systematic investigation and strong basis for the use of the GRAS fungus *A*. *oryzae* as a potential biocontrol agent for AFB1 contamination in food, and corroborates the expired patent for using certain strains of *A*. *oryzae* and *A*. *sojae* as biocontrol agents (US6027724A).

## Results

### Inhibition of AFB1 production by M2040

**Figure 2:**
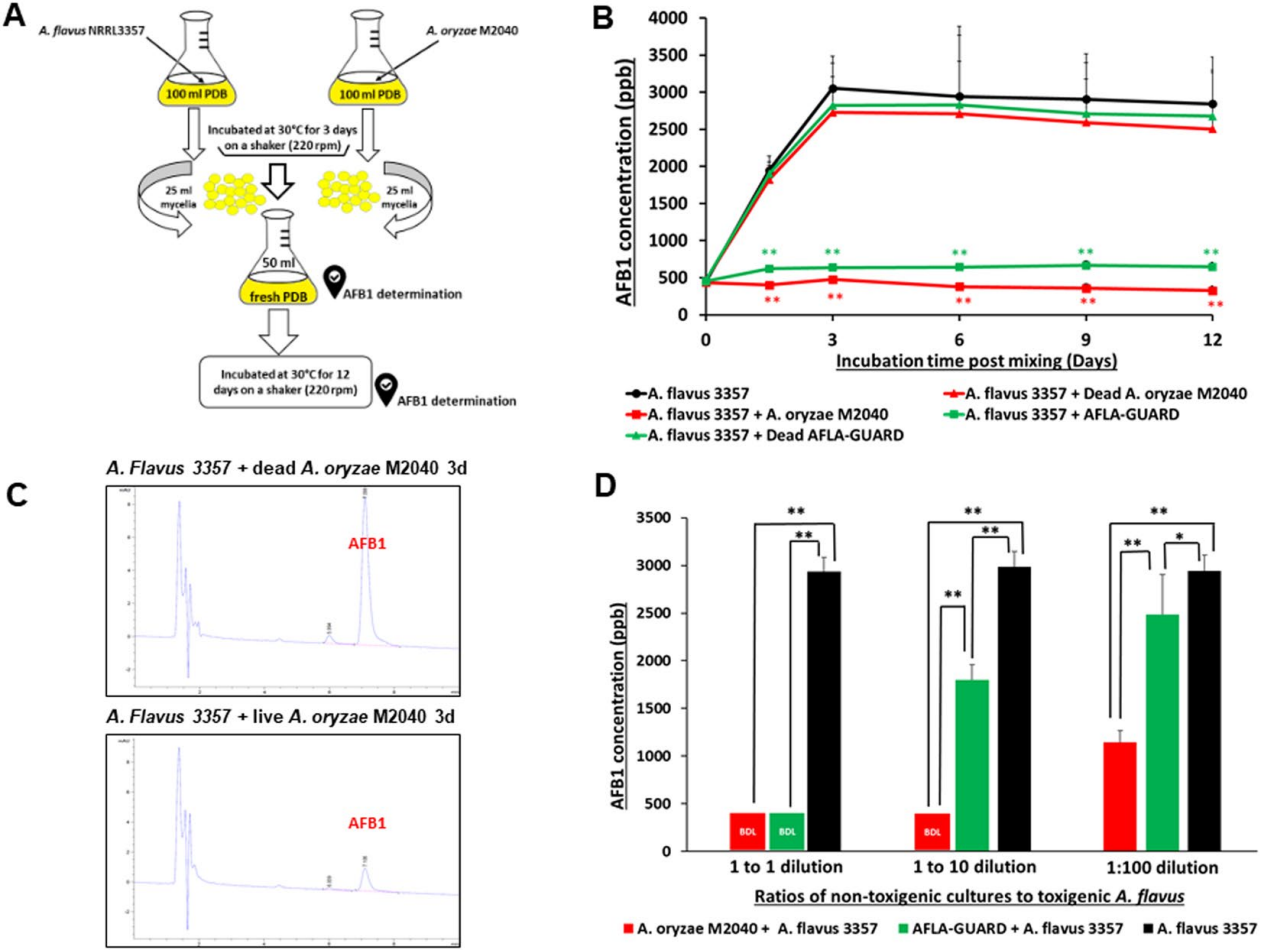
Inhibitory effects of *A*. *oryzae* M2040 on AFB1 production by *A*. *flavus*. (**A**) Experimental design. (**B**) Levels of AFB1 accumulation in a liquid co-culture media. *P < 0.05; **P < 0.01. (**C**) HPLC chromatograms of AFB1 at 3-day incubation of *A. flavus* vs dead and live M2040. Note the differences in the AFB1 peak size. (**D**) AFB1 accumulation and in peanut co-inoculated with M2040 and Afla-Guard and *A. flavus* NRRL3357 at different ratios.

To test the central hypothesis that M2040 inhibits AFB1 production, co-culture experiments of M2040 and *A*. *flavus* NRRL 3357, and Afla-Guard and *A*. *flavus* NRRL 3357 in PDB were performed as shown in Fig. 2A. We tested various media and found that PDB resulted in equal growth rates for M2040 and *A*. *flavus*, and high level production of AFB1. As controls, a set of the 3-day old cultures of M2040 and Afla-Guard were autoclaved (dead) and mixed with the 3-day old culture of *A*. *flavus* NRRL 3357. The mixed cultures were further incubated for up to 12 additional days and the amount of AFB1 was measured every 3 days. As shown in Fig. 2B, mixing live cells of both M2040 and Afla-Guard with *A*. *flavus* 3357 effectively blocked accumulation of AFB1 throughout the incubation. HPLC chromatograms of AFB1 in 3-day post mixing cultures clearly demonstrate the differences of AFB1 levels between co-culture of *A*. *flavus* 3357 with live and dead M2040 (Fig. 2C). AFB1 inhibition rates of M2040 were 98.8% and 100% at 3 and 12 days of incubation, respectively. Afla-Guard showed AFB1 inhibition rates of 93.0% and 94% at 3 and 12 days of incubation, respectively. Autoclaved (dead) cells of M2040 and Afla-Guard did not reduce AFB1 accumulation, resulting in accumulation up to 3000 ppb. These data indicate that M2040 can inhibit AFB1 production in PDB when co-cultured.

To corroborate the control of AFB1 contamination by M2040 on food matrix, we inoculated 1:1, 1:10, and 1:100 ratios of M2040 vs *A*. *flavus* NRRL 3357, and Afla-Guard vs *A*. *flavus* NRRL 3357 on peanuts and examined AFB1 levels at day 5 (Fig. 2D). At a 1:1 inoculation ratio, both M2040 and Afla-Guard blocked accumulation of AFB1 (Fig. 2D) compared to *A*. *flavus* NRRL 3357 alone (black bar). Surprisingly however, at 1:10 ratio (10% of a biocontrol strain), M2040 completely inhibited AFB1 accumulation while Afla-Guard allowed AFB1 accumulation to reach ~1,750 ppb. Furthermore, even at 1:100 ratio (1% of biocontrol strain), M2040 led to over 61% inhibition of AFB1 accumulation (Fig. 2D), whereas Afla-Guard allowed only about 15% inhibition compared to *A*. *flavus* NRRL 3357 alone. Collectively, these data indicate that M2040 has a very strong biocontrol potential that is comparable or superior to Afla-Guard.

### Quantification of *A*. *flavus* displacement by M2040

**Figure 3:**
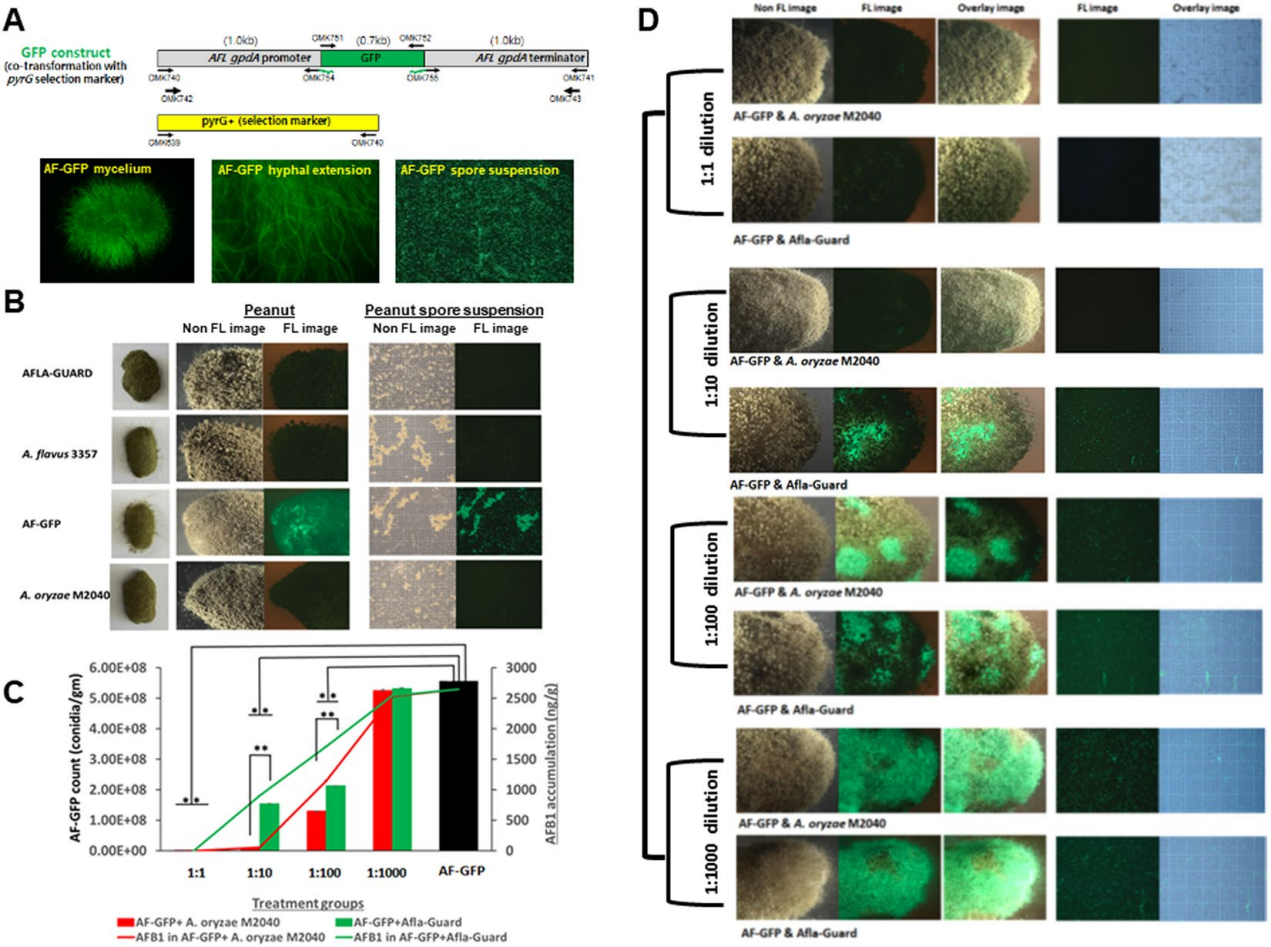
Quantitation of *A*. *flavus* displacement by *A*. *oryzae* M2040 and Afla-Guard on peanuts. (**A**) The GFP construct and 5 day old culture of AF-GFP showing highly fluorescent mycelia, hyphae, and conidial suspension. (**B**) Fluorescence (FL) and non-fluorescence images representing inoculation of control groups observed at 5 days of incubation. (**C**) AF-GFP conidial count and AFB1 accumulation in peanut samples co-inoculated with varying ratios of M2040 or Afla-Guard. *P < 0.05; **P < 0.01. (**D**) Fluorescence (FL) and non-fluorescence images of peanuts representing the treatment groups observed at 5 days of incubation. Photographs were taken in a dark room with a 1-2s exposure time.

In the aforementioned experiments AFB1 inhibition experiments, it was impossible to distinguish the target and control strains when they were mixed due to their morphological similarities. Thus, in order to quantify the growth rates of isolates in co-culture, we generated several GFP labeled *A*. *flavus* NRRL 3357 strains by co-transformation, and confirmed that these transformants produced AFB1 similar to wildtype. GFP levels of one strain named AF-GFP is depicted in Fig. 3A,B.

With the successful generation of AF-GFP, we performed co-inoculation experiments with AF-GFP and M2040, and AF-GFP with Afla-Guard on peanuts with 1:1, 1:10, 1:100, and 1:1,000 ratios. As shown in Fig. 3B–D, when co-inoculated with AF-GFP at a ratio of 1:1, both M2040 and Afla-Guard completely blocked AFB1 accumulation (100% inhibition rate), and essentially no AF-GFP spores were detected at 5 days of incubation (Fig. 3D, top panel). Importantly, M2040 exhibited 99% and 65% inhibition rates at 1:10 and 1:100 ratio, respectively, whereas Afla-Guard showed inhibition rates of 60% and 51% at ratio of 1:10 and 1:100, respectively. At 1:1000 ratio, both M2040 and Afla-Guard failed to inhibit AFB1 accumulation. Importantly, the recovery of AF-GFP spores and AFB1 levels were proportional throughout these experiments. At 1:1000 ratio, the peanuts were fully covered by AF-GFP (Fig. 3D, bottom panel). This experiment resulted in the first quantitative measure for *A*. *flavus* displacement by biocontrol strains, and suggests that M2040 can outcompete a toxigenic *A*. *flavus* strain even when present at only 1%.

### Cell-free culture broth of M2040 inhibits AFB1 accumulation

**Figure 4:**
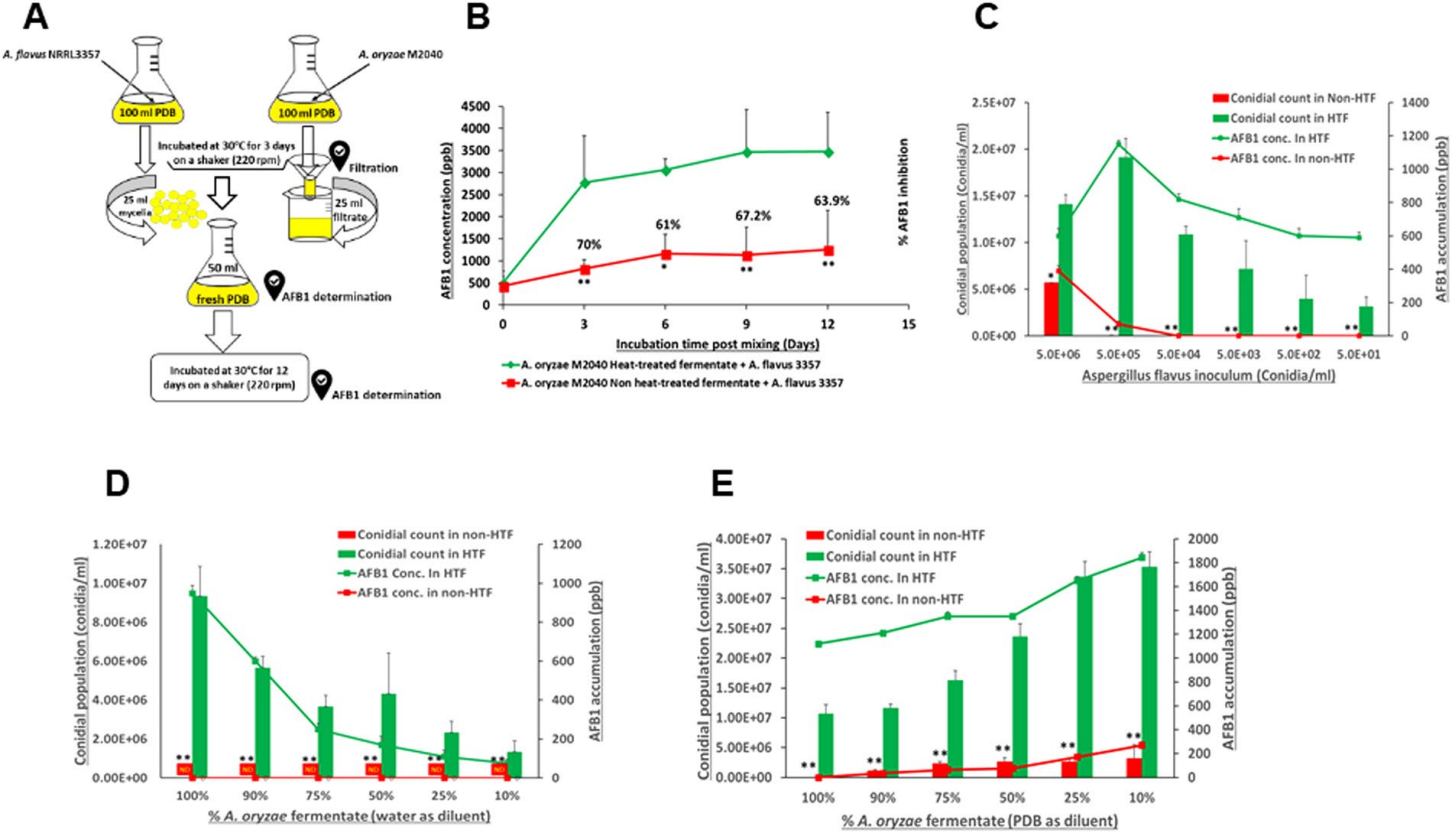
Effects of cell-free culture filtrate of *A*. *oryzae* M2040. (**A**) Experimental design. (**B**) Time course of the AFB1 accumulation in a mixed liquid of M2040 cell-free culture fermentate and *A. flavus* mycelial cells. *P < 0.05. **P < 0.01. (**C**) Conidial numbers and AFB1 production in HT and non HT M2040 fermentate inoculated with different conidial numbers of *A. flavus*. **P < 0.05. **P < 0.01. (**D**) Conidial count and AFB1 production in different concentrations of HT and non HT *A*. *oryzae* fermentate inoculated with 5 × 105 *A. flavus* conidia/ml. *P < 0.05. **P < 0.01. ND: Conidia were not detected under a microscope. Fermentate was diluted in sterile distilled water. (**E**) Conidial count and AFB1 production in different concentrations of HT and non HT M2040 fermentate inoculated with 5 × 10^5^ *A. flavus* conidia/ml. **P < 0.05. **P < 0.01. Fermentate was diluted in fresh PDB.

To test whether M2040 secretes unknown compound(s) into medium that confer AFB1 inhibition, we filter-sterilized (0.45 μm filter) the 8-day old culture of M2040 grown in PDB, and combined the cell-free culture with 3 day old culture of *A*. *flavus* 3357 and 50 ml of fresh PDB (Fig. 4A). As a control, a set of M2040 PBD culture filtrates were autoclaved (designated as heat-treated) and mixed with the 3 day old *A*. *flavus* culture. Autoclaving abolished the inhibitory effects of the cell-free culture (Fig. 4). Levels of AFB1 in the mixed medium were determined at 3, 6, 9 and 12 days post mixing. M2040 non-heat-treated culture filtrate was able to inhibit AFB1 accumulation at the inhibition rate of 60–70% compared to the control (heat-treated) group even at 25% levels (25 ml + 75 ml culture and PDB) (Fig. 4B).

To test the extent of the cell free culture filtrates ability to inhibit propagation and AFB1 production in *A*. *flavus*, we inoculated varying numbers (5 million to 50) of *A*. *flavus* NRRL 3357 spores into 2 mL of 100% culture filtrates, non-heat-treated, and heat-treated groups, and quantified the recovery of *A*. *flavus* spores and AFB1 levels (Fig. 4C). Non-heat-treated cell-free culture of M2040 effectively inhibited *A*. *flavus* growth and propagation as indicated by low conidial counts compared to the control group. It is important to note that active M2040 culture filtrate could effectively block the growth and AFB1 production even with 500,000 inoculated *A*. *flavus* spores (Fig. 4C). The maximum dilution levels of M2040 filtrate in water and PDB were further examined for inhibition of 500,000 *A*. *flavus* spores. As shown in Fig. 4D,E, the non-heat-treated culture fermentate in concentrations of 10%, 25%, 50%, 75%, and 100% (V:V in water or PDB) significantly inhibited *A*. *flavus* NRRL 3357 spore recovery and AFB1 production. These results indicate that the food grade (PDB) culture broth of the GRAS fungus M2040 can be effectively used as a safe agent to control *A*. *flavus* contamination and AF production.

### Whole genome sequencing and comparative analyses of M2040 and Afla-Guard

In order to verify that M2040 is indeed an *A*. *oryzae* strain lacking the ability to produce AFs, genomic DNA of M2040 was isolated, and genome sequencing was performed as previously described^24^. Though a draft genome sequence of Afla-Guard is available^25^ we sequenced its genome to obtain sufficient and comparable coverage for our comparative analysis. These genomes were also used to investigate potential genetic differences between *A*. *oryzae* M2040 and *A*. *flavus* Afla-Guard that may underlie their varying ability to inhibit AF production and proliferation of AF producing isolates. The M2040 and Afla-Guard genomes were both sequenced to >50X coverage and assembled into 1,479 and 1,766 scaffolds, with cumulative assembly sizes of 37.9 and 36.4 Mb, and N50 values of 135.8 Kb and 47.4 Kb, respectively. *In silico* gene prediction yielded 11,782 and 11,489 gene models for the M2040 and Afla-Guard genomes, respectively. Using antiSMASH 3.0, 46 and 42 secondary metabolic gene clusters were predicted in the M2040 and Afla-Guard genomes, respectively.

#### Phylogenetic analysis

**Figure 5:**
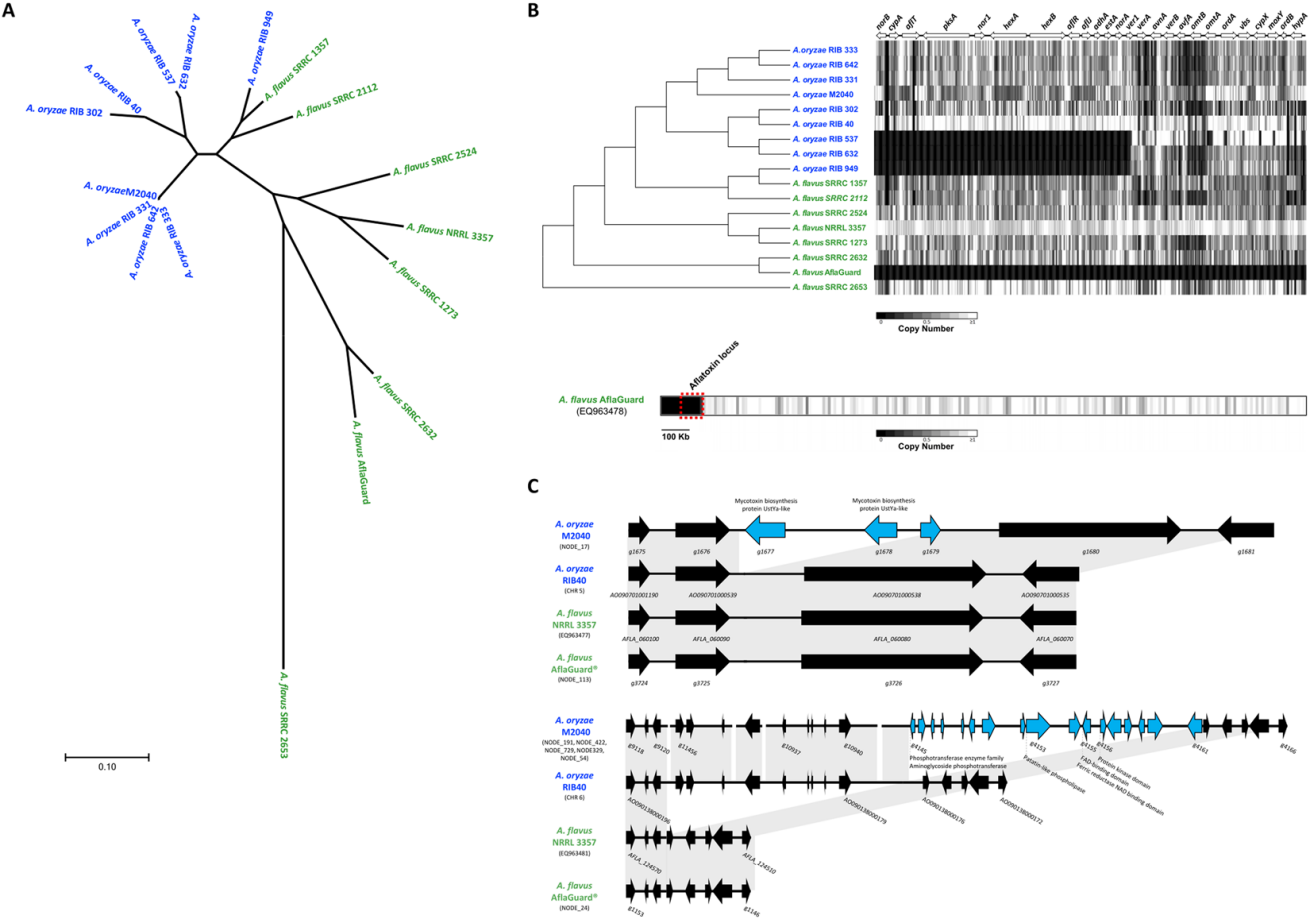
Comparative genome analyses of *A*. *oryzae* M2040 and Afla-Guard. (**A**) Phylogenetic relationship of *A*. *oryzae* and *A. flavus* isolates. An unrooted phylogeny was generated using the Maximum Likelihood method from 305,543 SNPs across the entire genome. Branch lengths represent the number of substitutions per site. All bootstrap values were ≥94%. Blue and green taxa labels represent *A*. *oryzae* and *A. flavus*, respectively. (**B**) Deletion profiles in the AF gene cluster. The chromosomal architecture of the AFB1 gene cluster relative to the *A. flavus* NRRL 3357 genome is shown above the heatmap, where arrows represent genes, and their orientations represents the direction of transcription. The heatmap represents copy number estimates for each non overlapping 100 bp bin across the AF gene cluster. Black and white represent copy numbers of 0 and ≥1, respectively. Bottom bar shows the Afla-Guard heatmap depicting deletions relative to the AF gene cluster containing *A. flavus* NRRL 3357 EQ963478 scaffold. Windows represent copy number estimates for each non-overlapping 10 kb bin across the scaffold. The chromosomal region containing the AF cluster is outlined with a red box. (**C**) Genome architecture of examples M2040 lineage specific genes clusters. Microsynteny of regions covering a three gene (top) and 17 gene (bottom) cluster unique to the M2040 genome in comparison to *A*. *oryzae* RIB 40, *A. flavus* NRRL 3357 and Afla-Guard. For each cluster arrows represent genes, and their orientations represents the direction of transcription. Genes colored black are conserved in at least 2 isolates, while genes colored light blue are unique to the M2040 genome. Gray blocks represent genomic regions exhibiting sequence similarity between isolates. Chromosome, or scaffold identifiers containing these loci are listed under each isolate. Gene identifiers are listed for each gene in panel A, and for the range of genes in panel.

The phylogeny of the 17 *A*. *oryzae* and *A*. *flavus* genomes was inferred using 305,543 whole-genome single nucleotide polymorphisms (SNPs: Fig. 5A). Consistent with earlier work^26^, our analysis suggests that the *A*. *oryzae* isolates, including *A*. *oryzae* M2040, are monophyletic and that *A*. *flavus* SRRC 1357 and SRRC 2112 show a closer relationship to *A*. *oryzae* than to other *A*. *flavus* isolates (Fig. 5A). Our results suggest that *A*. *oryzae* M2040 is extremely closely related to three Japanese sake derived isolates of *A*. *oryzae*, while *A*. *flavus* Afla-Guard is most closely related to *A*. *flavus* SRRC 2632 (Fig. 5A); a strain capable of producing cyclopiazonic acid, AFB1, and AFB2.

#### AF gene cluster variation

Using a read depth approach, we estimated copy number for each non-overlapping 100 bp bin across the AF biosynthetic gene cluster. *A*. *oryzae* M2040 possesses a number of deletions, including the intergenic region between the divergently transcribed *norB* and *cypA* genes^27^. *norB* is an aryl alcohol dehydrogenase, and *cypA* is a cytochrome P450 monooxygenase^28^. Both genes are involved in aflatoxin G formation. Isolates with this deletion do not express *norB* or *cypA*^26^. The entire AF biosynthetic gene cluster is deleted in *A*. *flavus* Afla-Guard (Fig. 5B). Closer examination of the *A*. *flavus* Afla-Guard genome reveals a ~155 Kb deletion beginning at the AF gene cluster and extending to the end of the chromosome (Fig. 5C).

#### Identification of *A*. *oryzae* M2040 lineage specific genes

Using a conservative BLAST based approach, lineage specific genes were identified in *A*. *oryzae* RIB 40, *A*. *oryzae* M2040, *A*. *flavus* NRRL 3357, and *A*. *flavus* Afla-Guard, with special focus given to *A*. *oryzae* M2040. We identified 111, 58, 140, and 111 lineage specific genes in the *A*. *oryzae* M2040, *A*. *oryzae* RIB 40, *A*. *flavus* NRRL 3357, and *A*. *flavus* Afla-Guard genomes, respectively. PFAM domains were predicted for 55 of the 111 *A*. *oryzae* M2040 genes. Interestingly, we identified two genes encoding proteins with heterokaryon incompatibility domains, one gene encoding a protein with an aminoglycoside phosphotransferase domain which confers resistance to various aminoglycosides, as well as several other genes encoding transporters, transcription factors, and protein kinases (Table 1).

**Table 1:** PFAM and InterPro annotation for *A*. *oryzae* M2040 lineage specific genes. The *A*. *oryzae* M2040 lineage specific genes and predicted domains.

Scaffold	Gene ID	PFAM Domain	InterPro Classification
NODE_14	g1437	Carbon-nitrogen hydrolase	Carbon-nitrogen hydrolase
NODE_17	g1677	Domain of unknown function (DUF3328)	Mycotoxin biosynthesis protein UstYa-like
NODE_17	g1678	Domain of unknown function (DUF3328) (x2)	Mycotoxin biosynthesis protein UstYa-like (x2)
NODE_35	g3027	Ankyrin repeat (x2)	Ankyrin repeat (x2)
		Ankyrin repeats (many copies)	AAA + ATPase domain
		AAA domain	Ankyrin repeat-containing domain
		Ankyrin repeats (3 copies)	
NODE_54	g4145	Phosphotransferase enzyme family	Aminoglycoside phosphotransferase
NODE_54	g4153	Patatin-like phospholipase	Patatin-like phospholipase domain
NODE_54	g4155	FAD-binding domain	FAD-binding 8
		Ferric reductase NAD binding domain	Ferric reductase, NAD binding domain
NODE_54	g4156	Protein kinase domain	Protein kinase domain
NODE_54	g4157	Protein of unknown function (DUF3723)	Protein of unknown function DUF3723
NODE_54	g4161	Protein of unknown function (DUF3435)	Protein of unknown function DUF3435
NODE_73	g5191	WD domain, G-beta repeat (x8)	WD40 repeat (x8)
NODE_193	g9143	SET domain	SET domain
NODE_194	g9174	AAA domain (x2)	
NODE_194	g9176	Argonaute linker 1 domain	Piwi domain
		Piwi domain	Argonaute, linker 1 domain
		PAZ domain	PAZ domain
		Helicase conserved C-terminal domain	Helicase, C-terminal
		N-terminal domain of argonaute	Protein argonaute, N-terminal
		Dicer dimerisation domain	Dicer dimerisation domain
		Ribonuclease III domain (x2)	Ribonuclease III domain (x2)
NODE_289	g10541	Protein kinase domain	Protein kinase domain
		Ulp1 protease family, C-terminal catalytic domain	Ulp1 protease family, C-terminal catalytic domain
NODE_290	g10546	Man1-Src1p-C-terminal domain	Man1-Src1p-C-terminal domain
NODE_326	g10914	Ferric reductase like transmembrane component	Ferric reductase transmembrane component-like domain
		FAD-binding domain	FAD-binding 8
NODE_326	g10915	Phosphorylase superfamily	Nucleoside phosphorylase domain
NODE_326	g10916	Protein kinase domain	Protein kinase domain
NODE_341	g11019	Protein of unknown function (DUF3645)	Protein of unknown function DUF3645
		Protein of unknown function (DUF3638)	Protein of unknown function DUF3638
NODE_369	g11222	Heterokaryon incompatibility protein (HET)	Heterokaryon incompatibility
NODE_371	g11231	Shwachman-Bodian-Diamond syndrome (SBDS) protein	Ribosome maturation protein SBDS, N-terminal
		ATP synthase alpha/beta chain, C terminal domain	ATP synthase, alpha subunit, C-terminal
		ATP synthase alpha/beta family, beta-barrel domain	ATPase, F1/V1/A1 complex, alpha/beta subunit, N-terminal domain
		ATP synthase alpha/beta family, nucleotide-binding domain	ATPase, F1/V1/A1 complex, alpha/beta subunit, nucleotide-binding domain
NODE_371	g11232	V-type ATPase 116 kDa subunit family	V-type ATPase, V0 complex, 116 kDa subunit family
NODE_378	g11269	Man1-Src1p-C-terminal domain	Man1-Src1p-C-terminal domain
NODE_378	g11273	Methyltransferase domain	Methyltransferase type 11
NODE_384	g11303	Heterokaryon incompatibility protein (HET)	Heterokaryon incompatibility
NODE_404	g11391	ATPase family associated with various cellular activities (AAA)	ATPase, AAA-type, core
NODE_416	g11434	Glycosyl hydrolase family 10 (x2)	Glycoside hydrolase family 10 domain (x2)
NODE_417	g11436	Glycosyl hydrolases family 32 N-terminal domain	Glycosyl hydrolase family 32, N-terminal
NODE_417	g11437	Sugar (and other) transporter	Major facilitator, sugar transporter-like
NODE_437	g11494	Alcohol dehydrogenase GroES-like domain	Alcohol dehydrogenase, N-terminal
		Zinc-binding dehydrogenase	Alcohol dehydrogenase, C-terminal
NODE_437	g11495	Eukaryotic elongation factor 5 A hypusine, DNA-binding OB fold	Translation elongation factor, IF5A C-terminal
		Elongation factor P (EF-P) KOW-like domain	Translation elongation factor, KOW-like
NODE_450	g11526	Cation transporter/ATPase, N-terminus	Cation-transporting P-type ATPase, N-terminal
NODE_450	g11527	MAC/Perforin domain	Membrane attack complex component/perforin (MACPF) domain
NODE_454	g11536	Ankyrin repeats (3 copies) (x3)	Ankyrin repeat-containing domain (x3)
		Ankyrin repeats (many copies)	
		Fungal specific transcription factor domain	Transcription factor domain, fungi
		AAA domain	AAA + ATPase domain
NODE_468	g11564	Protein of unknown function (DUF3435)	Protein of unknown function DUF3435
NODE_468	g11565	Protein kinase domain	Protein kinase domain
NODE_472	g11572	Protein of unknown function (DUF3435)	Protein of unknown function DUF3435
NODE_476	g11576	NmrA-like family	NmrA-like domain
NODE_479	g11582	Glycosyltransferase family 25 (LPS biosynthesis protein)	Glycosyl transferase, family 25
NODE_479	g11583	Glycosyltransferase sugar-binding region containing DXD motif	Glycosyltransferase, DXD sugar-binding motif
NODE_486	g11594	Ankyrin repeats (3 copies) (x2)	Ankyrin repeat-containing domain (x2)
NODE_486	g11595	Glycosyl transferase family 8	Glycosyl transferase, family 8
NODE_494	g11605	Phosphotransferase enzyme family	Aminoglycoside phosphotransferase
NODE_498	g11612	NACHT domain	
NODE_501	g11617	Clostridium epsilon toxin ETX/Bacillus mosquitocidal toxin MTX2	Clostridium epsilon toxin ETX/Bacillus mosquitocidal toxin MTX2
NODE_513	g11632	Haloacid dehalogenase-like hydrolase	
		Cation transporting ATPase, C-terminus	Cation-transporting P-type ATPase, C-terminal
NODE_541	g11662	T5orf172 domain	Bacteriophage T5, Orf172 DNA-binding
NODE_573	g11683	LysM domain (x3)	LysM domain (x3)
NODE_578	g11688	Sodium/hydrogen exchanger family	Cation/H+ exchanger
NODE_612	g11709	Sugar (and other) transporter	Major facilitator, sugar transporter-like
NODE_654	g11732	Phosphorylase superfamily	Nucleoside phosphorylase domain
NODE_691	g11743	Peptidase family M49	
NODE_713	g11751	BTB/POZ domain	BTB/POZ domain
NODE_860	g11779	E1-E2 ATPase	

Genes containing 2, 3, and 8 identical PFAM or InterPro domain are denoted as x2, x3, and x8, respectively. Genes g1679, g4146, g4147, g4148, g4149, g4150, g4151, g4152, g4154, g4158, g4159, g4160, g9173, g9175, g9177, g10449, g10540, g10542, g10543, g10544, g10545, g10547, g10913, g10943, g10944, g10945, g10946, g10947, g11018, g11020, g11270, g11271, g11272, g11307, g11438, g11487, g11503, g11504, g11566, g11588, g11660, g11673, g11674, g11681, g11682, g11699, g11707, g11714, g11716, g11729, g11733, g11736, g11760, g11765, g11777, and g11780 contained no predicted PFAM or InterPro classification.

In *A*. *oryzae* M2040, 63% of lineage specific genes were found in clusters of two or more genes, with an average cluster size of ~4 genes and the largest cluster containing 17 genes. Of note, we identified a cluster of three lineage specific genes in *A*. *oryzae* M2040 in which two of the genes contain mycotoxin biosynthesis protein UstYa-like domains (Fig. 5C). This protein domain is involved in the production of toxic cyclic peptides. Additionally, we identified a 17 gene cluster with varying genome architecture between *A*. *oryzae* M2040, *A*. *oryzae* RIB 40, and the two *A*. *flavus* isolates (Fig. 5C). Most of these genes encode proteins annotated as hypothetical proteins in other organisms, though BLAST searches against the RefSeq non-redundant protein database, PFAM domain prediction, and InterPro classification revealed proteins annotated as phosphotransferase family protein, patatin/phospholipase A2-related, tyrosine-protein kinase, and casein kinase family protein.

## Discussion

The use of atoxigenic *A*. *flavus* to control AFs was demonstrated in 1990s (Peter Cotty USDA Agricultural Research Service) in Arizonian cotton fields^18^. However, studies dated back to 1965 found that AF levels can be reduced by co-cultivating toxigenic *A*. *flavus* strains with certain fungi or bacteria^29,30^. This biocontrol strategy is currently the most widely used method for reducing contamination levels of AFs in some crops. Nevertheless, there are many challenges facing this strategy at both short and long-term^31,32^. One of the major drawbacks of the biocontrol strategy is a potential risk of introducing a heavy dose of *A*. *flavus* strains that could alter the soil microbiome populations especially with global warming. The inherent diversity of *A*. *flavus* populations makes a biocontrol strategy more difficult because *A*. *flavus* populations differ in their abilities to produce AFs and other toxic secondary metabolites^31^. To address these potential issues, we tested the potential use of *A*. *oryzae* M2040 as a biocontrol agent.

Prior to the beginning of the studies, we tested the inhibitory activity of 10 *A*. *oryzae* strains and found that M2040 most significantly inhibited AF production and *A*. *flavus* growth. Our data indicates that AFB1 is not detected in PDB when equal number of spores of *A*. *oryzae* and *A*. *flavus* are co-inoculated. Therefore, in order to test the precise inhibitory effects, we cultured each strain separately for 3 days and mixed equal volume (25 mL each) of the fungal cell aggregates (mycelia) in new PDB medium (50 mL) and quantified submerged growth and AFB1 production. Additionally, Afla-Gurad^®^ was selected as a positive control based on its effective use *in vitro* and in the field to control AFs contamination. Both live cells of *A*. *oryzae* and Afla-Gurad^®^ significantly inhibited AFB1 accumulation over a range of laboratory conditions indicating that fungal cell-cell interactions may act as an additional control factor mediating the inhibitory process.

To track the competitive effect of nontoxigenic strains, we co-inoculated M2040 and Afla-Guard at varying inoculum levels with a transgenic *A*. *flavus* NRRL 3357 (AF-GFP) strain expressing GFP on peanut samples. AFB1 was measured as well. Previous experiments indicated that Afla-Guard can result in more than 85% reduction in AFs^33,34^. At 1:1 ratio, the AF-GFP fluorescence in the co-inoculated peanut kernels was significantly reduced, and AFB1 was not detected by when co-cultured with M2040 or Afla-Guard. Since the inoculum size is the same for both non-toxigenic and toxigenic strains, the faster growing isolate (biocontrol strains) should outcompete the other isolate (toxin-producing strain). However, when both biocontrol agents were further diluted to 10% levels, M2040 clearly showed superior reduction (87%) in total AFB1 accumulation compared to Afla-Guard (60.6%). Abbas *et al*.^21^ reported 58% and 83% in AF reduction when they applied the non-aflatoxigenic *A*. *flavus* K49 strain in the two-corn fields at a ratio of 10-fold dilution. However, when K49 was applied at equal inoculum size with toxigenic one, AFs were reduced by 86% and 96%. It has been hypothesized that displacement of the toxigenic strains occurs simply by the biocontrol strains superior ability to better sequester nutrients. However, using different biocontrol strains has resulted in variable reduction rates of AFs, suggesting that other unknown factors may be involved for the biocontrol mechanism such as the production of extracellular compounds that might inhibit AFB1 production. M2040 showed significant activity for AFB1 reduction (50%) even at 100-fold dilution compared to Afla-Guard (26%), however, at 1000-fold dilution, both biocontrol agents resulted in AFB1 reduction of less than 10%.

When cell free culture fermentate of M24040 was tested for inhibiting the mycelial growth and AFB1 production of *A*. *flavus*, it was found to be highly effective against AFB1 production by reducing levels by more than 70%. Furthermore, our broth microdilution experiments revealed that the fermentate was found to be highly effective in inhibiting conidial germination and AFB1 production. The inhibitory activity of the culture fermentate was observable even at 10% concentration and these effects were abolished by autoclaving, but not by treating with proteinase K. These results suggest the non-protein nature of the active substances in the fermentate.

To better understand the phenotypic differences between M2040 and Afla-Guard through a broader evolutionary context, we sequenced the genomes of both isolates. This analysis yielded several key findings. First, we note that Afla-Guard is nested within an aflatoxigenic clade of *A*. *flavus*, and most closely related to the clinical isolate *A*. *flavus* SRRC 2632 which is capable of producing AF and cyclopiazonic acid (CPA) (Fig. 5A). Our read depth analysis confirms that the Afla-Guard genome lacks the ability to produce AF and CPA because of a ~155 Kb deletion spanning the gene clusters encoding both of these secondary metabolites^35^ (Fig. 5B). Additionally, our phylogenetic analysis revealed that M2040 is nearly identical to three Japanese strains isolated from miso (RIB 331 and RIB 333) and sake (642) (Fig. 5A).

Genome sequencing of M2040 and Afla-Guard also allowed us to conduct comparative genomic analysis to identify potential gain-of-function genes that might be associated with the inhibitory ability of M2040. We hypothesized that certain genes involved in unknown anti-fungal products may curtail growth and AF production of *A*. *flavus*. We identified 111 lineage-specific genes in the M2040 genome, including several genes that may be involved in biosynthesis of toxin-like products. For instance, gene *g11617* was annotated by a “Clostridium epsilon toxin ETX/Bacillus mosquitocidal toxin MTX2” domain (Table 1). BLAST searches against the NCBI non-redundant protein database reveal the presence of this gene in the *A*. *oryzae* BCC7051 genome^36^, along with only two other significant hits to the *Talaromyces cellulolyticus* Y-94 genome^37^ (E-value = 7e^−17^) and the *Botrytis cinerea* BcDW1 (E-value = 8e^−12^)^38^. We also identified two neighboring genes (*g1677* and *g1678*) that have the Interpro classification “Mycotoxin biosynthesis protein UstYa-like” (Fig. 5C). *ustYa* is an oxidase involved in the production of ustiloxins and homologs have been identified in *A*. *flavus* NRRL 3357^39^ and *A*. *oryzae* RIB 40. However, homologs to the *A*. *oryzae* M2040 genes *g1677* and *g1678* are not present in *A*. *oryzae* RIB 40, *A*. *flavus* NRRL 3357, or *A*. *flavus* Afla-Guard, and BLAST searches against the NCBI non-redundant protein database show a patchy distribution across Ascomycota including *A*. *oryzae* BCC7051^36^, *A*. *bombycis*^40^, *A*. *udagawae*^41^, *Xylona heveae*^42^, *Penicillium oxalicum*^43^, and *Penicillium nordicum*^44^. Moving forward, defining the transcriptional landscape of M2040 in co-culture will allow us to further narrow in on the genes involved in inhibiting AF production and growth.

In conclusion, our studies raise the idea of potentially using a food grade *A*. *oryzae* strain as a potent biocontrol agent to reduce *A*. *flavus* growth and AF contamination. Additionally, *A*. *oryzae* cell free PDB fermentate could be employed as a valuable biocontrol agent. To the best of our knowledge, our report is the first study systematically showing the advantages of using GRAS fungus, and its fermentate to control *A*. *flavus* growth and AFs contamination. These results, along with further studies, will eventually provide a GRAS product(s) that can be used as a natural anti-fungal and anti-AF agent.

## Materials and Methods

### Fungal strains and culture conditions

The aflatoxigenic strain *A*. *flavus* NRRL3357 was used as a high AFB1 producer^45^. *A*. *flavus* NRRL3357 labeled with green fluorescent protein (AF-GFP) was developed and used in this study. Bright fluorescence was observed for mycelia and hyphae of the AF-GFP (Fig. 3A). *A*. *flavus* NRRL 21882 (Afla-Guard) and *A*. *oryzae* M2040 (isolated from Meju, Korea) were used as non-aflatoxigenic strains. All strains were maintained on potato dextrose agar (PDA) medium (containing 4 g potato starch, 20 g glucose, and 15 g agar in 1 L of distilled water) at 4 °C. To prepare inoculum, *Aspergillus* cultures were grown on PDA for 7 days at 30 ± 2 °C. Spores were harvested from individual cultures on PDA using 0.1% Tween-80 solution. Asexual spores (conidia) were counted with a hemocytometer and numbers were adjusted to 5 × 10^7^ conidia/mL with water. Fungal spore suspensions were stored at 4 °C and used within 1 week of preparation.

### Generation of GFP labeled *A*. *flavus* NRRL 3357 strains

The oligonucleotides used in this study are listed in Table 2. Double joint PCR (DJ-PCR) was used to generate AF-GFP strains^46^. To generate the PCR amplicon of the GFP Open Reading Frame (ORF), the primer pair OMK751; OMK752 was used from pFNO3 (vectors provided by Dr. P. N Keller) plasmid DNA. Both 5′ and 3′ flanking regions of the glyceraldehyde phosphate dehydrogenase (*gpdA*) gene was amplified from genomic DNA of *A*. *flavus* NRRL 3357 using OMK740; OMK754 and OMK755; OMK741. The final DJ-PCR of AF-GFP construct was amplified with OMK742; OMK743. The *A*. *flavus pyrG*^+^ marker was amplified with the primer pair OMK639; OMK640. The final DJ-PCR construct and the *A*. *flavus pyrG*^+^ marker amplicons were co-introduced into *A*. *flavus* NRRL3357.5 (*pyrG*^-^). Protoplasts were generated using the Vinoflow FCE lysing enzyme as described (Novozymes)^47^. Fungal transformants were isolated and confirmed by PCR followed by restriction enzyme digestion^46^. At least three independent AF-GFP strains were isolated and confirmed for AFB1 production.

**Table 2:** Oligonucleotides used in this study.

Name	Sequence (5′ → 3′)	Purpose
OMK740	TCACTGAAAAAGAGCTAAGACTA	5′ flanking of AFL *gpdA*
OMK741	TCCCATGACAGTGTCTTCGT	3′ flanking of AFL *gpdA*
OMK742	ACCCCAGTACAGTTTCATGCAA	5′ nest of AFL *gpdA*
OMK743	TTGCGCAGAAGCCTAGACAAGTC	3′ nest of AFL *gpdA*
OMK751	ATGAGTAAAGGAGAAGAACTT	5′ GFP ORF
OMK752	TTTGTATAGTTCATCCATGC	3′ GFP ORF
OMK754	*AAGTTCTTCTCCTTTACTCAT*TGTTTAGATGTGTCTGTTG	3′ AFL *gpdA* with GFP tail^a^
OMK755	*TGGCATGGATGA ACTATACAAA*AAGTCATACCTAACAAGTGCT	5′ AFL *gpdA* with GFP tail^a^
OMK639	TCGAGAGATGAGGGCTGCCAGCA	5′ *AFL pyrG* marker
OMK640	CAGAAGAAAAGGATGATCAATAC	3′ *AFL pyrG* marker

^a^Tail sequence is in italic.

### Reagents and chemicals

AFB1 standard was purchased from Sigma-Aldrich (U.S.). Water, methanol, acetonitrile chloroform were purchased from Fisher Chemical (U.S.). All solvents were of HPLC-grade. Membrane filters (47 mm × 0.45 μm) and syringe filters (13 mm × 0.2 μm) were obtained from Millipore (U.S.).

### HPLC analysis of AFB1

#### Extraction of AFB1 from liquid culture media

AFB1 was extracted from submerged media by liquid-liquid extraction. Briefly, 2 ml of the fungal culture broth was mixed with equal volume of chloroform in 15-ml centrifuge tube and vortexed for 60 sec, left at room temperature for 5 min, then vortexed again for 60 sec. The mixture was then centrifuged for 5 min at 5000 *g*. Two ml of the lower layer was transferred to a new glass vial. The chloroform extracts were evaporated to complete dryness under a gentle stream of air. The dried extracts were reconstituted with 1 ml methanol. All samples were filtered into HPLC vials through 0.2 μm syringe filter prior to HPLC analysis.

#### Measurement of conidia and AFB1 from peanut

Extraction of AFB1 from peanuts was performed as described^48^ with slight modifications. Two peanut cotyledons were placed in a 50-ml Falcon tube containing 5 ml of 0.1% Tween 80 and each tube was vortexed thoroughly. To check the conidial number, 1 mL was transferred from each sample into an Eppendorf tube and spores were counted. Next, 5 mL of acetone was added to the remaining samples, followed by shaking for 15 min in a rotary shaker at 150 rpm. Samples were kept standing for 5 min at room temperature, 5 ml of chloroform was then added, and samples were agitated for 15 min at 150 rpm. Samples were left to stand for an additional 5 min at room temperature. The organic lower layer was collected by centrifugation of samples for 10 min at 5000 *g*, transferred to a new tube and dried under gentle stream of air. The dried extracts were reconstituted with 1 ml methanol. All samples were filtered into HPLC vials through 0.22 μm disposable syringe filter prior to HPLC analysis.

#### Chromatographic conditions

Samples were analyzed for AFB1 using a model 1100 HPLC system consisting of a degasser, an autosampler, a quaternary pump, and a diode array (DAD) detector (Agilent). Samples were eluted at a wave length of 365 nm with a mobile phase of H_2_O:CH_3_OH:CH_3_CN (50:40:10) at a flow rate of 0.8 ml/min. The mobile was degassed and filtered through membrane filter (47 mm × 0.45 μm) prior to use. The separation was performed *via* a Zorbax Eclipse XDB-C18 4.6 mm × 150 mm, 3.5 μm column. The injection volume was 10 μl. AFB1 peaks area were recorded and integrated using ChemStation software (Agilent). The limit of AFB1 detection was 1 ppb.

### Determining the effect of *A*. *oryzae* M2040 on AFB1 production in submerged culture

Conidia (5 × 10^7^) of *A*. *oryzae* M2040 and *A*. *flavus* NRRL 3357 were separately inoculated into PDB (100 ml) in 250 ml Erlenmeyer flask and incubated at 30 ± 2 °C with shaking at 220 rpm. After 3 days, 25 ml of *A*. *oryzae* and 25 ml of *A*. *flavus* mycelia were transferred to a new 250 ml Erlenmeyer flask containing 50 ml of fresh PDB. This co-cultured mixture (100 ml) was incubated for 12 days under the same culture conditions mentioned above. AFB1 concentration in the culture medium was assayed every three days. The following three controls were used in this study; 1) Co-cultured mixture of *A*. *flavus* and dead cells of *A*. *oryzae* M2040 heat-treated by autoclaving at 121 °C and 15 psi for 20 min, 2) *A*. *flavus* NRRL 3357 only, 3) Co-cultured mixture of *A*. *flavus* NRRL 3357 and *A*. *flavus* Afla-Guard. All treatments were tested in triplicate flasks and the experiment was performed three time.

### Determining the effect of *A*. *oryzae* M2040 on *A*. *flavus* growth and AFB1 production in peanuts

We tested the effect of varying inoculation ratios of *A*. *oryzae* M2040, *A*. *flavus* Afla-Guard, and our toxigenic GFP-labeled strain of *A*. *flavus* NRRL 3357 (AF-GFP) on AFB1 accumulation and sporulation 5 days after inoculation. This experiment was performed in triplicate for each treatment group and repeated twice.

#### Preparation of peanut samples

Peanut infection procedure was performed as described previously^49^ with some modifications. Mature peanut seeds were obtained by local market and prepared by removing the exterior layer. Two cotyledons were separated, and the embryo was removed gently. Then, cotyledons (0.5 g) were surface sterilized by placing them in beaker containing 0.05% NaClO in sterile water for 2 min. Then the cotyledons were washed by placing them in a new beaker containing sterile distilled water for 1 min, followed by a 10-second wash with 70% ethanol in a new beaker. A final washing step with sterile distilled water for 2 min was performed to ensure complete removal of detergents. The cotyledons were dried completely for at least 2 hours under aseptic condition until the time of infection.

#### Peanut infection

Peanut cotyledons were allocated into four treatment groups. Group 1 was inoculated with 100,000 spores of AF-GFP alone, which served as a control. Group 2 was co-inoculated with 1:1 (10^5^:10^5^), 1:10 (10^4^:10^5^), 1:100 (10^3^:10^5^), and 1:1000 (10^2^:10^5^) of *A*. *oryzae* M2040 relative to 10^5^ spores of AF-GFP. Group 3 was inoculated with 1:1 (10^5^:10^5^), 1:10 (10^4^:10^5^), 1:100 (10^3^:10^5^), and 1:1000 (10^2^:10^5^) of *A*. *flavus* Afla-Guard relative to 10^5^ spores of AF-GFP. Group 4 was treated with water (mock inoculation). For all treatments, 10 peanut cotyledons were used for each plate in triplicate. Cotyledons were placed in petri dishes lined with 3 pieces of moist filter paper and a water reservoir (lid of a 50 ml centrifuge tube containing 2 ml of sterile water) to maintain high humidity. Cotyledons were incubated for 5 days at 30 °C.

#### Determination of conidia number and AFB1 accumulation

AFB1 was extracted from peanut cotyledons as described above and calculated for all treatment groups. In order to determine the extent of AF-GFP growth in peanut and the percentage of toxigenic strain reduction, conidia of AF-GFP were counted in all groups by hemocytometer and the microscopy images were taken using a Zeiss Axio Observer D4 Fluorescence Microscope with Achromat S 1, 5x FWD 28 mm lenses (Carl Zeiss, Germany).

### Determining the effects of cell free culture fermentate of *A. oryzae* M2040 on *A*. *flavus* growth and AFB1 production

The effects of cell free culture fermentate on AFB1 production by *A*. *flavus* was studied in 250 ml Erlenmeyer flasks under submerged culture condition. Conidia (5 × 10^7^) of *A*. *oryzae* and *A*. *flavus* were inoculated separately in 100 ml PDB and incubated at 30 ± 2 °C with shaking at 220 rpm. After 3 days, 25 ml of *A*. *oryzae* M2040 cell free culture fermentate was transferred to a new flask containing 50 ml of fresh PDB and, at this time, 25 ml of *A*. *flavus* mycelia was also added to this flask. This mixture was incubated for 12 days under the same culture conditions mentioned above and the AFB1 concentrations in the culture medium were assayed every 3 days of incubation. Autoclaved (heat-treated) cell free culture fermentate was used as a control. All treatments were tested in triplicate and the experiment was repeated twice.

#### Production of the cell free culture fermentate

Conidia (5 × 10^8^) of *A*. *oryzae* M2040 were inoculated into 1000 mL of PDB in a 2 L Erlenmeyer flasks and incubated at 30 ± 2 °C for 8 days with shaking at 220 rpm. The mycelia were separated from the culture broth by filtration with four layers of Miracloth (MilliporeSigma) and the cell free culture fermentate was obtained by filtering through a 0.2 μm PES filter unit (Thermo Scientific, USA). The fermentate was kept at 4 °C and used within one month of production. Portions of the fermentate were treated with proteinase-K (BDH Biochemicals).

#### Testing the effect of cell-free culture fermentate on the growth and germination of A. flavus at different conidia counts

Using a 24-well microdilution plate, 2 ml of the fermentate was loaded into 6 wells and *A*. *flavus* conidia were kept in the first well at 5 × 10^6^ conidia/ml final concentration. Ten-fold serial dilutions were made to 5 × 10^1^ conidia/ml. Heat treated fermentate served as a control. The plate was incubated at 30 ± 2 °C for 5 days. Samples were harvested, conidia counted with a hemocytometer, and AFB1 levels were estimated. All treatments were tested in triplicate wells and repeated at least three times.

#### Testing different concentrations of cell-free culture fermentate on the growth and germination of A. flavus

Cell-free culture fermentate was diluted in sterile distilled water or PDB to give final concentration (v/v) of 100%, 90%, 75%, 50%, 25%, and 10%. Heat-treated cell-free culture fermentate was prepared as a control. Two ml of treatment groups were loaded into the well and *A*. *flavus* was inoculated in all wells at a final concertation of 5 × 10^5^ conidia/ml. The plate was incubated at 30 ± 2 °C for 5 days. Samples were harvested, conidia were counted with a hemocytometer, and AFB1 levels were quantified. All treatments were tested in triplicate wells and repeated at least three times.

### Genome Sequencing and Assembly of *A. oryzae* M2040 and Afla-Guard

Genomic DNA was extracted as previously described^24^. A paired-end 152-bp Illumina library was constructed from genomic DNA and sequenced at ProteinCT (Madison, Wisconsin). Illumina sequence data were first deduplicated using Tally^50^. Next, trim_galore (https://www.bioinformatics.babraham.ac.uk/projects/trim_galore/) was used to trim reads at bases with quality scores < 30. Trimmed read pairs, with at least 1 read < 50 bp were discarded. Trim_galore was also used to removed residual adapter sequences from reads, using the conservative parameter “stringency = 1”. Lastly, this set of deduplicated, quality trimmed, and adapter trimmed reads were error corrected using SPAdes version 3.10.0^51^, resulting in a high-quality dataset of 15,376,723 paired-end reads, representing ~115X coverage for M2040, and 8,042,928 paired-end reads, representing ~53X coverage for Afla-Guard. Both genomes were assembled using SPAdes version 3.10.0^51^ using the “careful” mismatch mode and *k-mers* sizes of 25, 35, 45, 55, 65, 78, 85, and 95. antiSMASH 3.0 was used to predict secondary metabolic encoding gene clusters in the *A*. *oryzae* M2040 and *A*. *flavus* Afla-Guard genomes^52^.

### Phylogenetic analysis

We examined the evolutionary relationships of *A*. *oryzae* M2040, Afla-Guard, and previously sequenced isolates^26^ using an alignment of 305,543 SNPs collected across the entire genome. SNPs were collected using the Phylogenetic and Molecular Evolution (PhaME) analysis tool on assembled genomes. All genome assemblies were performed using SPAdes version 3.10.0^51^ as described above. The *A*. *oryzae* RIB 40 and *A*. *flavus* NRRL 3357 genomes were obtained from FungiDB^53^. The *A*. *oryzae* RIB 40 genome was used as the reference during SNP prediction with PhaME. A phylogenetic tree was constructed using the Maximum Likelihood method based on the Hasegawa-Kishino-Yano model^54^ with 100 bootstrap replicates in MEGA7^55^.

### AF gene cluster variation analysis

A number of deletions responsible for limiting AFB1 production have been previously characterized^27,56^. We used a read depth approach to better characterize large scale deletions in the AF biosynthetic gene cluster in the *A*. *oryzae* M2040 and *A*. *flavus* Afla-Guard genomes. For all isolates, reads were mapped against the reference *A*. *flavus* NRRL 3357 genome using the “sensitive” pre-set parameters in bowtie2^57^. SAM alignment files were converted into sorted BAM format using the view and sort functions in SAMtools^58^. The SAMtools depth function was then used to estimate average coverage across the entire genome. Average coverage values for each non-overlapping 100 bp portion of the AF gene cluster were then divided by the average coverage across the entire genome to estimate copy number.

To further test the M2040’s inability to produce AFs, the strain was cultured in submerged and solid-state media. PDB and PDA are considered the best media for optimal AFs production. One milliliter (5 × 10^7^) of *A*. *oryzae* M2040 spore suspension was inoculated into 100 ml of medium and incubated for 10 days at 30 °C with shaking at 220 rpm. For testing the AFB1 production in solid media, 0.1 ml (5 × 10^6^) of *A*. *oryzae* M2040 spore suspension was streaked on PDA plates and incubated for 7 days at 30 °C. The amount of AFB1 in the liquid and solid culture medium was analyzed by HPLC and TLC.

### Identification of lineage specific genes

We conservatively identified lineage specific genes in *A*. *oryzae* M2040, *A*. *flavus* Afla-Guard, *A*. *oryzae* RIB 40^22^, and *A*. *flavus* NRRL 3357^59^. Gene models were predicted in the *A*. *oryzae* M2040 and *A*. *flavus* Afla-Guard genome assembly using Augustus v2.5.5^60^ with the following parameters: “strand = both”, “genemodel = complete”, and “species = aspergillus_oryzae”. Lineage specific genes were conservatively identified in each genome by pairwise BLAST searches of each isolate’s gene models against each genome^61^. Gene models with BLAST scores > 1e-6 were considered unique to each respective isolate. *phmmer* and *hmmscan* were used to annotate lineage specific genes^62^.

### Statistical analysis of the data

Statistical significance was determined using student’s *t*-test with a 2-tailed distribution. Difference was considered significant as P < 0.05. Error bars correspond to the standard deviation.

## Data Availability

The genome sequence data are available under the BioProject ID (PRJNA483302) and the SRA Run IDs (SRR7615261 for *A*. *oryzae* M2040 and SRR7615262 for *A*. *flavus* Afla-Guard). https://www.ncbi.nlm.nih.gov/Traces/study/?acc = SRP155606.
